# Minimal overall divergence of the gut microbiome in an adaptive radiation of *Cyprinodon* pupfishes despite potential adaptive enrichment for scale-eating

**DOI:** 10.1371/journal.pone.0273177

**Published:** 2022-09-16

**Authors:** Joseph Heras, Christopher H. Martin

**Affiliations:** 1 Department of Integrative Biology, University of California, Berkeley, Berkeley, CA, United States of America; 2 Museum of Vertebrate Zoology, University of California, Berkeley, Berkeley, CA, United States of America; University of Konstanz, GERMANY

## Abstract

Adaptive radiations offer an excellent opportunity to understand the eco-evolutionary dynamics of gut microbiota and host niche specialization. In a laboratory common garden, we compared the gut microbiota of two novel derived trophic specialist pupfishes, a scale-eater and a molluscivore, to closely related and distant outgroup generalist populations, spanning both rapid trophic evolution within 10 kya and stable generalist diets persisting over 11 Mya. We predicted an adaptive and highly divergent microbiome composition in the trophic specialists reflecting their rapid rates of craniofacial and behavioral diversification. We sequenced 16S rRNA amplicons of gut microbiomes from lab-reared adult pupfishes raised under identical conditions and fed the same high protein diet. In contrast to our predictions, gut microbiota largely reflected phylogenetic distance among species, rather than generalist or specialist life history, in support of phylosymbiosis. However, we did find significant enrichment of *Burkholderiaceae* bacteria in replicated lab-reared scale-eater populations. These bacteria sometimes digest collagen, the major component of fish scales, supporting an adaptive shift. We also found some enrichment of *Rhodobacteraceae* and *Planctomycetia* in lab-reared molluscivore populations, but these bacteria target cellulose. Overall phylogenetic conservation of microbiome composition contrasts with predictions of adaptive radiation theory and observations of rapid diversification in all other trophic traits in these hosts, including craniofacial morphology, foraging behavior, aggression, and gene expression, suggesting that the functional role of these minor shifts in microbiota will be important for understanding the role of the microbiome in trophic diversification.

## Introduction

Rapid evolutionary change can alter ecological processes which in turn change the course of evolutionary processes [1, 2]. This process is described as eco-evolutionary dynamics, which provides a framework for understanding the interplay between evolution and ecological interactions [3, 4]. The emergence of studies that focus on eco-evolutionary dynamics has provided insight for community assembly, ecological speciation, and adaptive radiations [3]. A better understanding of these eco-evolutionary dynamics can be applied to host-microbiota interactions, in which microbes and host relationships impact host performance and fitness [5–7]. The microbial community may also play a large role in ontogeny, immunity, physiology, and the ecology and evolution of the host [8–11].

Several studies have now examined gut microbiome diversification in an adaptive radiation of hosts, including fishes [6, 10, 12–14]. Similarity among host species microbiomes that recapitulates the evolutionary history of the host species is known as phylosymbiosis and is frequently the primary hypothesis in these studies [15, 16]. However, these studies rarely examine outgroups to the focal radiation in order to compare rates of microbiome divergence. Furthermore, phylosymbiosis [comparable to phylogenetic conservatism; 17] contrasts with the expectations of a rapid burst of phenotypic diversification during adaptive radiation, which would suggest that the microbiome within an adaptive radiation should diverge far more quickly than outgroup taxa due to rapid ecological divergence and specialization [18–22]. Thus, we predicted greater microbiome divergence within a recent adaptive radiation of trophic specialists than among outgroup generalist taxa.

An adaptive radiation of *Cyprinodon* pupfishes provides an excellent opportunity to test the relative roles of rapid trophic divergence and phylosymbiosis in shaping the gut microbiome. Pupfishes are found in hypersaline lakes and coastal areas throughout the Caribbean and Atlantic (most are allopatric) and within isolated desert pools and streams [23–25]. However, there are only two sympatric adaptive radiations of trophic specialists across this range [26]. One radiation is endemic to San Salvador Island, Bahamas, containing a generalist algivorous and detritivorous species, *Cyprinodon variegatus*, and two trophic specialist species, a molluscivore (durophage) *C*. *brontotheroides* and a scale-eater (lepidophage) *C*. *desquamator* [26–28; there is also a fourth intermediate scale-eating species not including in this study: 28]. Scale-eating and molluscivore niches are uniquely derived within this sympatric radiation on San Salvador Island relative to a generalist or omnivore diet of macroalgae and micro-invertebrates in all other *Cyprinodon* species spread across the Caribbean and desert interior of North America [23, 29], including the most closely related extant genus *Cualac* [26]. The adaptive radiation of *Cyprinodon* pupfishes on San Salvador Island is estimated to be around 10,000 years old based on the age of the hypersaline lakes on the island which filled with rising sea levels following the last glacial maximum [30–32]. In contrast, the most divergent generalist population in our study, the checkered pupfish *Cualac tessellatus*, occurs only in the El Potosí desert spring system in Mexico and last shared a common ancestor with *Cyprinodon* 11.2 Mya [33]. The two trophic specialist species on San Salvador Island are derived from a generalist ancestor and each shows signatures of adaptive introgression and the reassembly of standing genetic variation in generalist populations from across the Caribbean [24, 34]. Thus, this radiation provides an excellent opportunity to compare microbiome divergence within a sympatric adaptive radiation of trophic specialists nested within a large clade of generalist/omnivorous which have not substantially shifted their dietary niches over millions of years.

Despite extensive craniofacial and behavioral divergence between trophic specialists endemic to San Salvador Island there are very few fixed genetic differences between these species: there are only 157 fixed SNPs between molluscivores and scale-eaters out of 10 million segregating SNPs and only 87 deletions fixed in scale-eaters relative to molluscivores [34]. These are likely overestimates of fixed differences due to our smaller sample sizes of each species (approximately 30 per species). However, these fixed genetic differences may be driving differences in gut microbiome composition. Intriguingly, the only fixed coding indel detected so far in this system is a fixed deletion in all scale-eater populations of the fifth exon of the gene *gpa33* [34]. This is an oncogene expressed in the intestinal epithelium and mice knockouts display a range of inflammatory intestinal pathologies [35], suggesting it may play a role in the gut microbiota composition of scale-eaters. Overall genetic differentiation among species is minimal, even within the same lake, ranging from *F*_*st*_ = 0.1–0.3, suggesting that soft sweeps and allele frequency changes among ecotypes may also play a larger role in their adaptation to different diets.

We raised all species in our study in a common laboratory environment for at least one generation and fed them an identical commercial pellet diet for one month before sampling gut microbiomes. All pupfishes were fed pellet food throughout their lives after feeding exclusively on newly hatched brine shrimp for approximately the first month after hatching. Importantly, all species and outgroups were treated in exactly the same way during lab-rearing. We addressed the following questions: 1) Do gut microbial communities primarily reflect dietary specialization or phylogenetic distance among species? 2) Is there a microbiome signal associated with lepidophagy (scale-eating) or molluscivory? We found enrichment only for the microbial family *Burkholderiaceae* in our two independent scale-eating pupfish colonies. This is significant because members of this microbial family digest collagen, the major component of fish scales. Overall, we infer a minor but potentially adaptive shift in the scale-eater microbiome, even when rearing hosts in identical environments on identical non-scale diets.

## Materials and methods

### Sampling and preparation of gut microbiome samples

Colonies of *Cyprinodon* pupfishes were collected from two hypersaline lakes on San Salvador Island, Bahamas (Crescent Pond and Osprey Lake) and Lake Cunningham, Bahamas in March, 2018 and were reared for two generations in aquaria at the University of North Carolina at Chapel Hill and the University of California, Berkeley. One generalist population was collected in May, 2018 from Fort Fisher Estuary in North Carolina and wild fish raised in the lab for one year were used for this study because no lab-reared fish were yet available at sufficient size. All fish were collected and exported with research permits from the Bahamas Environmental Science and Technology (BEST) commission or the U.S. Fish and Wildlife Service. *Cualac tessellatus* eggs were provided by the Zoological Society of London and reared in the lab for two generations before used for the four samples in this study. Exact generation times are unknown for this captive colony, but likely exceeded ten generations in captivity. All samples, except for the recently collected NC population, came from first or second-generation captive-bred individuals reared in aquaria (40–80 L) at 5–10 ppt salinity (Instant Ocean synthetic sea salt) and between 23 to 30°C. Colonies were always kept isolated by species and location. Individuals used for this study were fed once daily *ad libitum* with a single commercial pellet food (New Life Spectrum Cichlid Formula, New Life International, Inc., Homestead, FL), containing 34% crude protein, 5% crude fat, and 5% crude fiber, for one month without exposure to any other food or tankmates. Before this period, individuals were reared on pellet food throughout their lives following approximately one month feeding exclusively on newly hatched baby brine shrimp (*Artemia* spp.) after hatching. All animal care and experiments were conducted under approved protocols and guidelines of the University of California, Berkeley Institutional Animal Care and Use Committee (AUP-2018-08-11373).

In total, forty fishes were euthanized in an overdose of MS-222 and the entire intestinal tract was immediately excised (Cyprinodontidae do not possess stomachs; [36]) for DNA extraction. Standard length (the distance from the tip of the snout on the fish to the base of the caudal fin), and gut length were measured for all samples (S1 Table), and analyzed with an ANCOVA. Five individuals (F_2_ generation) from each of three species (*C*. *variegatus*, *C*. *brontotheroides*, and *C*. *desquamator*) in two replicate lake populations from San Salvador Island were sampled (*n* = 30 total), Crescent Pond and Osprey Lake. Conspecific populations show minimal genetic differentiation (*F*_*st*_ = 0.1–0.3) between these lakes [29, 37], providing independent replicates of the ecotypes across two hypersaline lake environments. In addition, we included the following generalist pupfish species as outgroups to our study: *C*. *laciniatus* (F1 generation; Lake Cunningham, New Providence Island, Bahamas; *n* = 4), *C*. *variegatus* (F0 generation raised in the lab for one year; Fort Fisher, North Carolina, United States; *n* = 2) plus liver tissue as a tissue control to compare with the gut microbiome, and *Cualac tessellatus* (long-term captive colony; San Luis Potosí, Mexico, *n* = 4).

Each gut was divided into proximal and distal regions for all San Salvador Island samples to compare microbial composition between these regions. We subsampled the gut only for the San Salvador Island (our focal group) samples to determine if the microbiome composition differs throughout the intestine. The outgroup guts were not the primary focus of this study, therefore we sampled the entire gut for all outgroups. In addition, the microbial community was haphazardly sampled from aquaria water in two tanks which contained F2 individuals of Osprey Lake *C*. *variegatus* and Crescent Pond *C*. *variegatus*, for a control sample of the existing aquatic microbial community in the common garden lab environment (*n* = 2). These two samples were taken concurrently with the end of our sampling for this study. The Vincent J. Coates Genomics Sequencing Laboratory at the University of California, Berkeley also generated three controls, including a positive control and two no template controls (NTC). Microbial DNA extractions were performed in batches (stored on ice) immediately after intestinal dissections with the Zymobiomics DNA Miniprep Kit (Zymo Research, Irvine, CA).

### 16S amplicon sequencing of gut microbiomes

All extracted microbiome DNA samples were quantified with a Nanodrop ND-1000 spectrophotometer (range 4.2–474.9 ng/μl). All samples were then sent to the QB3 Vincent J. Coates Genomics Sequencing Laboratory at the University of California, Berkeley for automated library preparation and sequencing of 16S rRNA amplicons using an Illumina Mi-Seq v3 (600 cycle). As part of the QB3 library preparation, the Forward ITS1 (ITS1f)–CTTGGTCATTTAGAGGAAGTAA and Reverse ITS1 (ITS2)–GCTGGGTTCTTCATCGATGC primers [38] were used for DNA metabarcoding markers for fungi [38]. We removed all eukaryotic sequence reads from our analyses which included fungi reads. QB3 also used the following 16S rRNA primers for amplification of prokaryotes (archaea and bacteria): Forward 16S v4 (515Fb)– GTGYCAGCMGCCGCGGTAA, and Reverse 16S v4 (806Rb)– GGACTACNVGGGTWTCTAAT [39, 40].

### Bioinformatic analysis/quantification and microbial ecology assessment of samples

All 16S rRNA amplicon sequences were processed through qiime 2.0 [41] to identify microbe species and estimate abundances. Sequences from all 78 microbiome preps were imported into qiime 2 v. 2019.10.0. We used dada2 [q2-dada2 version 2019.10.0; 42] for modeling and correcting Illumina-sequenced amplicon errors, removing chimeras, trimming low quality bases, and merging of forward and reverse reads using the following parameters:–p-trunc-len-f 270 –p-trunc-len-r 210. The end product of dada2 is an amplicon sequence variant (ASV) table. We used the qiime alignment mafft software to align sequences alignment mask to filter non-conserved and highly gapped columns from the aligned 16S sequences [43]. Next, we used qiime phylogeny midpoint-root to root the phylogeny of our 16S amplicon sequences. Finally, we used qiime diversity alpha-rarefaction on all samples and we set the—p-max-depth to 10,000. We removed samples with 5,000 (only one sample fell below this threshold, *Cualac tessellatus*; S1 Fig) or less from our analyses as suggested [44, 45].

We compared the beta diversity (qiime emperor plot) of proximal and distal gut microbiomes of the San Salvador samples with a two-tailed paired *t*-test and found no significant differences between proximal and distal regions of the gut microbiome (*P* = 0.29). Therefore, we merged the proximal and distal samples for each individual from San Salvador Island, resulting in 48 samples, which included experimental controls and quality controls from the QB3 facility (S2 Table). There was no difference between the means of amplicon sequence reads in the foregut and the hindgut (paired t-test, *P* = 0.29). We also removed one *Cualac tessellatus* sample because of low read count (129 reads; S1 Fig).

**Fig 1:**
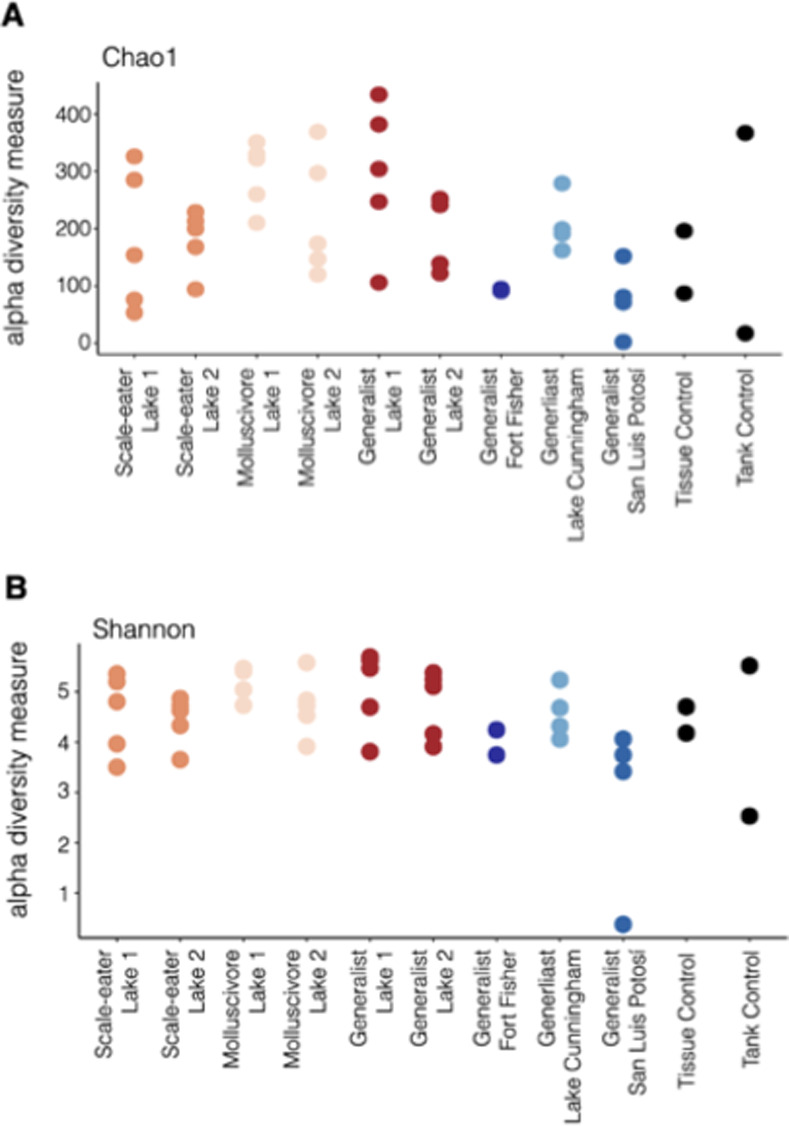
Alpha diversity of *Cyprinodon* pupfishes gut microbiomes based on parental location and diet type along with controls. Lake 1 indicates Crescent Pond and Lake 2 represents Osprey Lake, both located on San Salvador Island in the Bahamas. Alpha diversity is represented by (A) Chao1 and (B) Shannon diversity for the estimate of species richness from gut microbiomes from all fishes in this study.

We used the classifier Silva 132 99% 515F/806R (silva-132-99-515-806-nb-classifier) for training in identification of taxa from our samples. All qiime commands described above were completed in qiime 2 v. 2019.10.0. Afterwards the following files generated in qiime were used in r (v. 4.0.0) for further statistical analyses: table.qza, rooted-tree.qza, taxonomy.qza, and sample-metadata.tsv. We used the following R packages for further analyses: phyloseq v.1.32.0 [46] with the following functions: distance, plot_bar, plot_ordination, and plot_richness. We used ggplot2 v.3.3.2 [47] for creating plots that we generated from phyloseq. Before conducting any analyses, we removed the following taxa from our analyses, uncharacterized and Opisthokonta (eukaryotic sequences mainly due to fish 16S amplicons). We plotted alpha diversity by using the plot_richness function as part of phyloseq, and we plotted both Chao1 and Shannon’s diversities (Fig 1). For beta diversity, we used the plot_ordination function and non-metric multidimensional scaling (NMDS) based on Bray-Curtis distances among samples. We conducted a Permutational multivariate analysis of variance (PERMANOVA) based on Bray-Curtis similiarty matrix to test whether diet or species impact the fish gut microbiomes. This was done with adonis2 which is part of the vegan package (https://CRAN.R-project.org/package=vegan) with 9,999 permutations. We also tested for significance at group-level differences using multivariate homogeneity of groups dispersions with betadisper, which is also part of the vegan package. Hierarchical clustering was generated with the distance function along with hclust as part of fastcluster [48] using the average linkage clustering method. The plot_bar function in the phyloseq package was used to visualize relative abundance of taxa. In only our taxa plots, we removed abundance counts of less than 400 from our analyses to provide the prevalent taxa with higher abundances across samples [49]. We used ggplot2 to generate all figures [47]. We used the linear discriminant analysis effect size [lefse version 1.0; 50] algorithm to identify microbial taxa that were significantly enriched in each of our specialists (molluscivore and scale-eater) in comparison to all other samples. This analysis was used to determine the features (i.e. organisms, clades, operational taxonomic units) to explain differences in assigned metadata categories. We used the nonparametric factorial Kruskal-Wallis rank-sum test to detect taxa with significant differential abundances between specialist samples and all generalist samples (scale-eater versus generalist + molluscivore, molluscivore versus generalist + scale-eater). We then used a Wilcoxon test for all pairwise comparisons between taxa within each significantly enriched class to compare to the class level.

Lastly, we used generalized linear models (GLMs) in r to test the effects of diet (generalist, scale-eater, molluscivore), the fixed effect of location (Osprey Lake, San Salvador Island; Crescent Pond, San Salvador Island; Lake Cunningham, New Providence Island; Fort Fisher, North Carolina; and San Luis Potosí, Mexico), and their interaction on the response variables of principal coordinates axes 1 and 2. We used Principal Coordinates Analysis (PCoA) for only the GLMs to conducts these tests.

## Results

### Gut microbiome diversity and divergence among taxa and intestinal lengths

**Fig 2:**
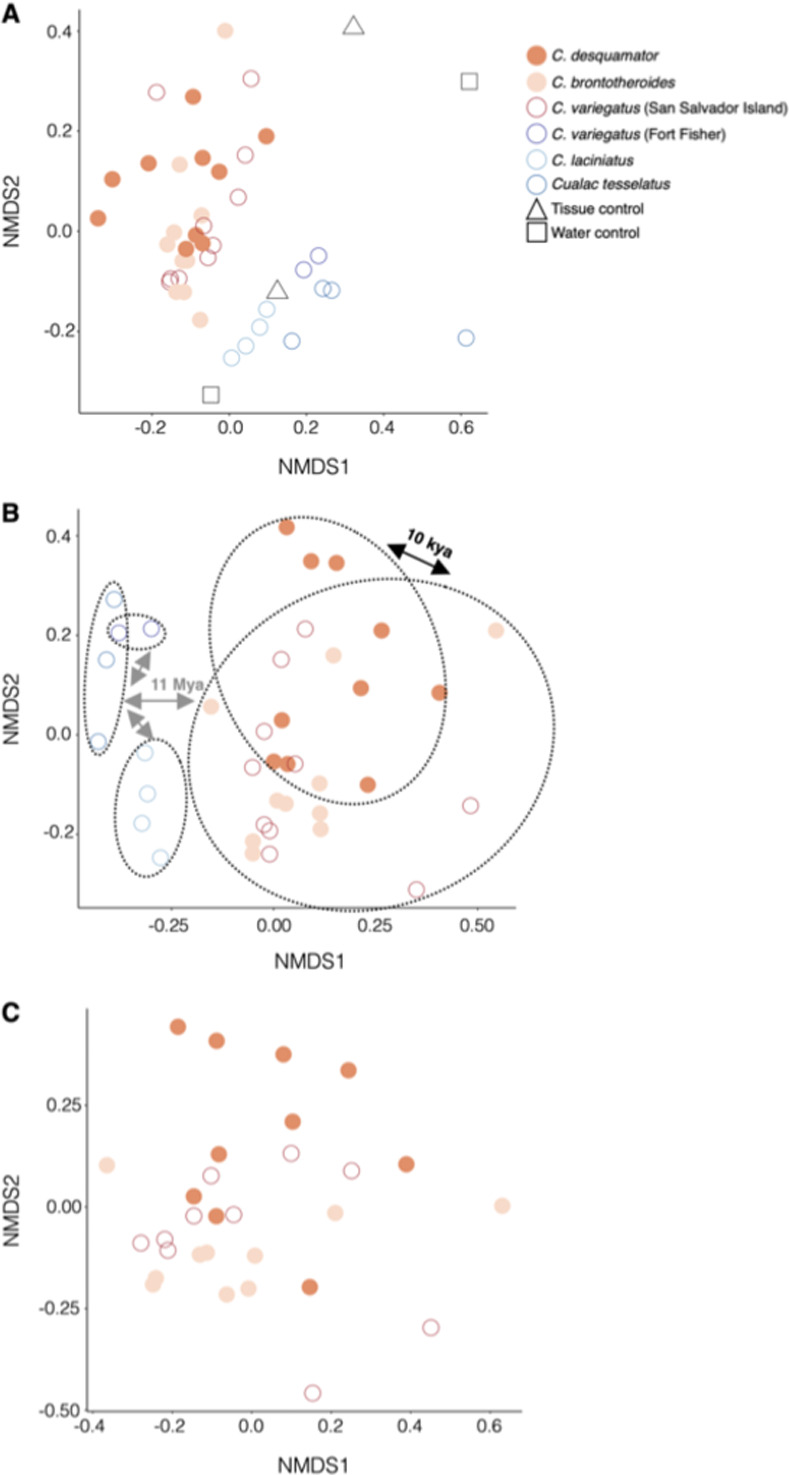
Non-metric multidimensional scaling (NMDS) plots of *Cyprinodon* pupfish gut microbiomes. A) NMDS plot based on all *Cyprinodon* pupfish gut samples labeled according to species and diet including controls (n = 44). Closed circles represent the two specialists (scale-eater and molluscivore) and open circles represent generalists. Open squares and triangles represent controls used in this study. B) Shows a NMDS plot of the three *Cyprinodon* pupfish species (F2 generation) from San Salvador Island and outgroup members gut microbiomes (n = 39). According to Echelle et al. [33], there is ~11.2 Mya phylogenetic divergence of *Cualac tessellatus* and *Cyprinodon* pupfish species, which you can see in the gray arrows and text. The black arrow indicates the 10 kya phylogenetic divergence of *C*. *desquamator* and the other *Cyprinodon* pupfish species from San Salvador Island. C) Lastly, a NMDS plot of only the San Salvador Island species gut microbiomes (n = 30).

We sequenced a total of 11,152,147 reads across all samples (S2 Table). We identified 5,174 bacterial taxa in 48 samples. Similar to other ray-finned fishes [51], proteobacteria is the predominant microbial taxon (S3 Fig). We did not find any significant differences among species in Chao1 or Shannon diversity indices (Kruskal-Wallace [pairwise], *P* > 0.05; Fig 1). Twenty-one San Salvador Island pupfishes clustered together relative to the three outgroup generalist species, indicating strong host phylogenetic signal associated with overall microbiome diversity (S4 Fig). We also noticed that eight San Salvador Island pupfishes were more dissimilar than the outgroup generalists; however, this may have been due to limited microbial material sampled from these individuals. Throughout the dendrogram, we noticed that within the San Salvador pupfishes, the two specialists (molluscivore and scale-eater) clustered with San Salvador generalists (Figs 2 and S4 and S5). Water and tissue controls were scattered throughout the NMDS plots but were clearly distinct from *Cyprinodon* microbiome samples with the exception of one tissue control that clustered near the outgroup species, possibly due to contamination during dissections (Fig 2). The ordination stress values for our NMDS plots were 0.191, 0.207, 0.200 for all samples in the study including controls (Fig 2A), San Salvador Island and outgroup species gut microbiomes (Fig 2B), and San Salvador Island species gut microbiome (Fig 2C), respectively. Using PERMANOVA, we found significant differences among gut microbiome communities by diet (df = 1, F = 2.210, *P* = 1e-04, and betadisper *P* = 0.512); and by species (df = 5, F = 2.196, *P* = 1e-04, and betadisper *P* = 0.000166).

Multiple regression analyses of the effects of dietary specialization (generalist, scale-eater, or molluscivore) and the fixed effect of population origin (two different lakes on San Salvador Island, Lake Cunningham, North Carolina, and El Potosí) on PCoA axes 1 and 2 confirmed that population origin and scale-eating had a significant effect on microbiome divergence along both axes (Axis.1: scale-eater *P* = 0.001 with 52.8% proportion of variance; Axis.2: scale-eater *P* = 0.018 with 10.3% proportion of variance). However, when evaluating residual deviance compared to the null deviance for both generalized linear models, both showed no differences when using a chi-squared test (glm, *P* > 0.05). In our comparison of the pupfish gut lengths in our study, we found no significant difference in gut lengths after controlling for specimen size (S2 Fig; ANCOVA with covariate of log-transformed SL; *F*_*5*,*33*_ = 0.916, *P* = 0.483).

### Linear discriminate analyses of trophic specialist microbiota

**Fig 3:**
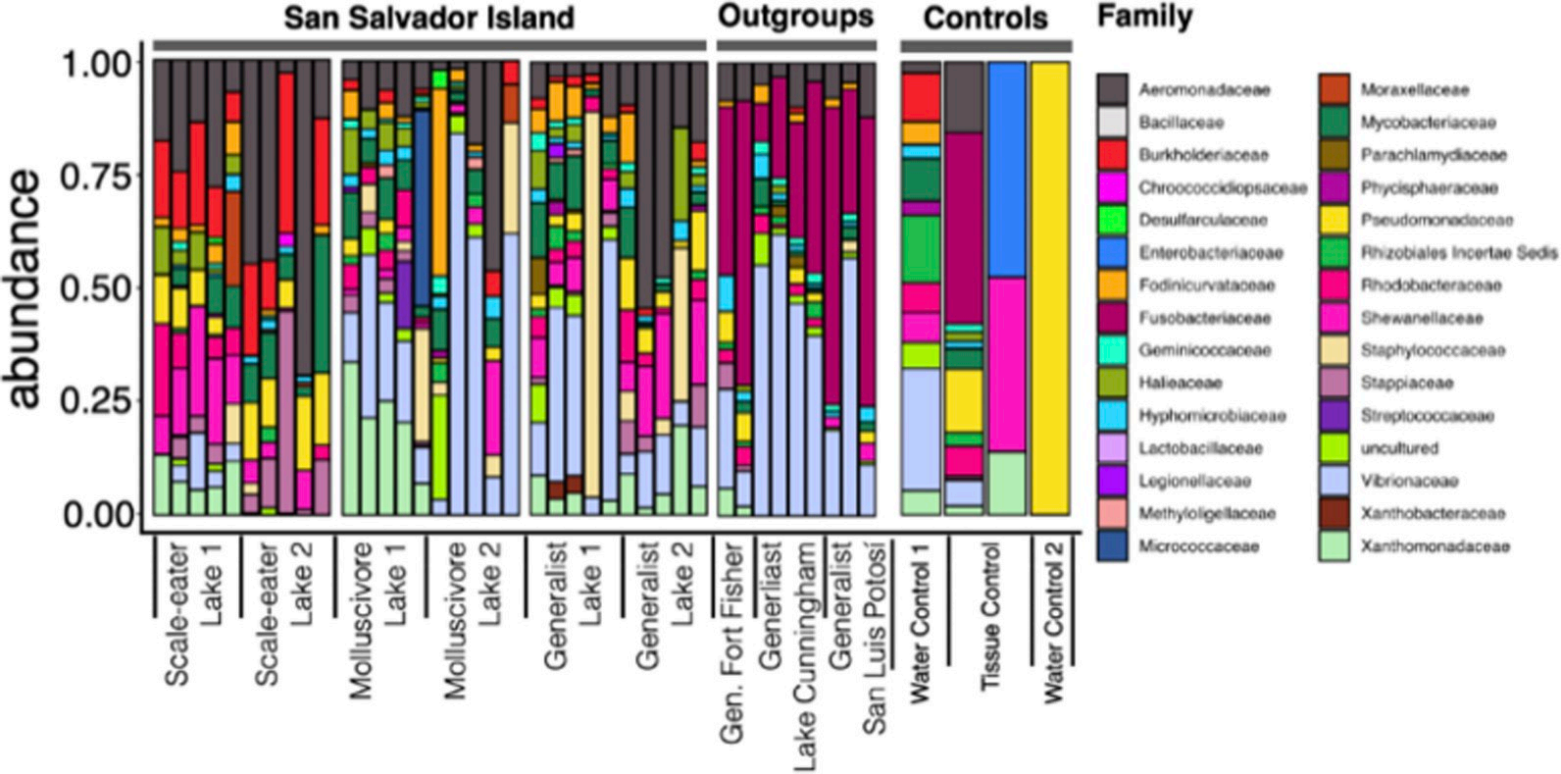
Taxa plot of the microbial composition of the *Cyprinodon* gut microbiome and controls. Bars show proportions (relative abundance) of taxa at the family level per individual gut microbiome. Lake 1 indicates Crescent Pond and Lake 2 represents Osprey Lake, both located on San Salvador Island in the Bahamas. Taxa which contained uncharacterized and Opisthokonta (eukaryotic sequences) were removed and taxa with a count of 400 or greater were represented. Taxa were grouped according to species and location (controls included).

**Fig 4:**
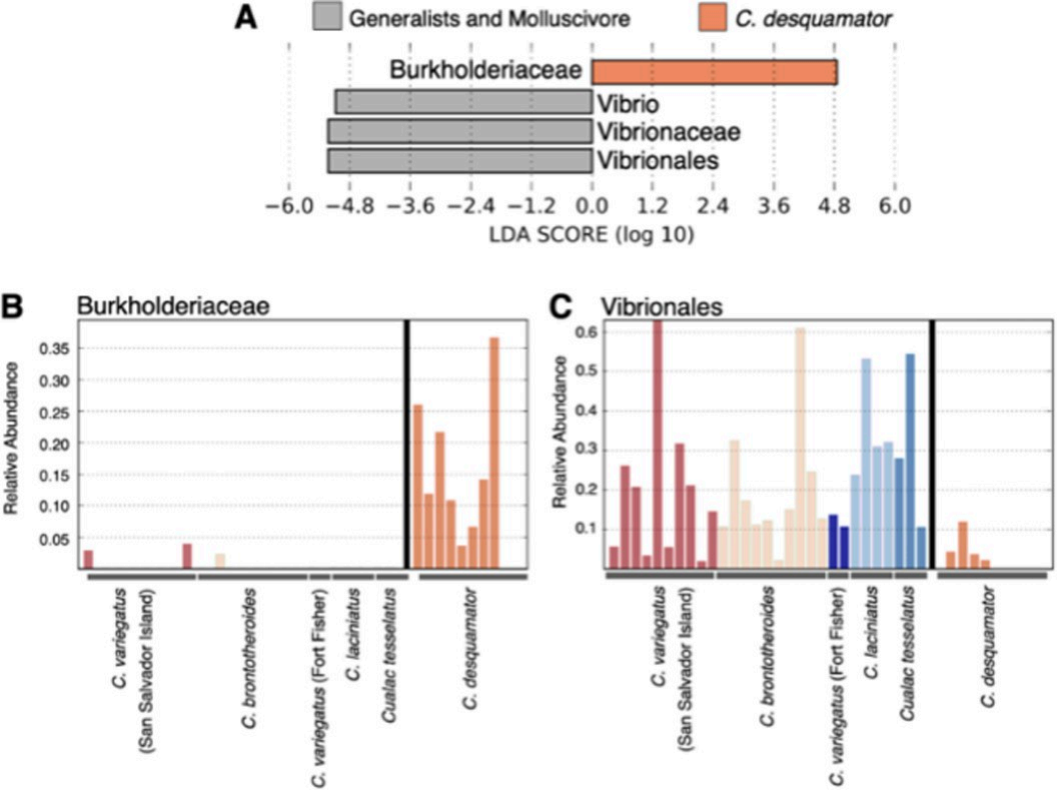
Linear discriminate analysis between *Cyprinodon desquamator* (scale-eater) and non-scale eaters. A) Log scores of the top four dominant loadings on lefse discriminate axis separating scale-eaters from all other pupfish samples. B) Relative abundance of the family *Burkholderiaceae* and the order C) *Vibrionales* among all pupfish gut microbiomes.

**Fig 5:**
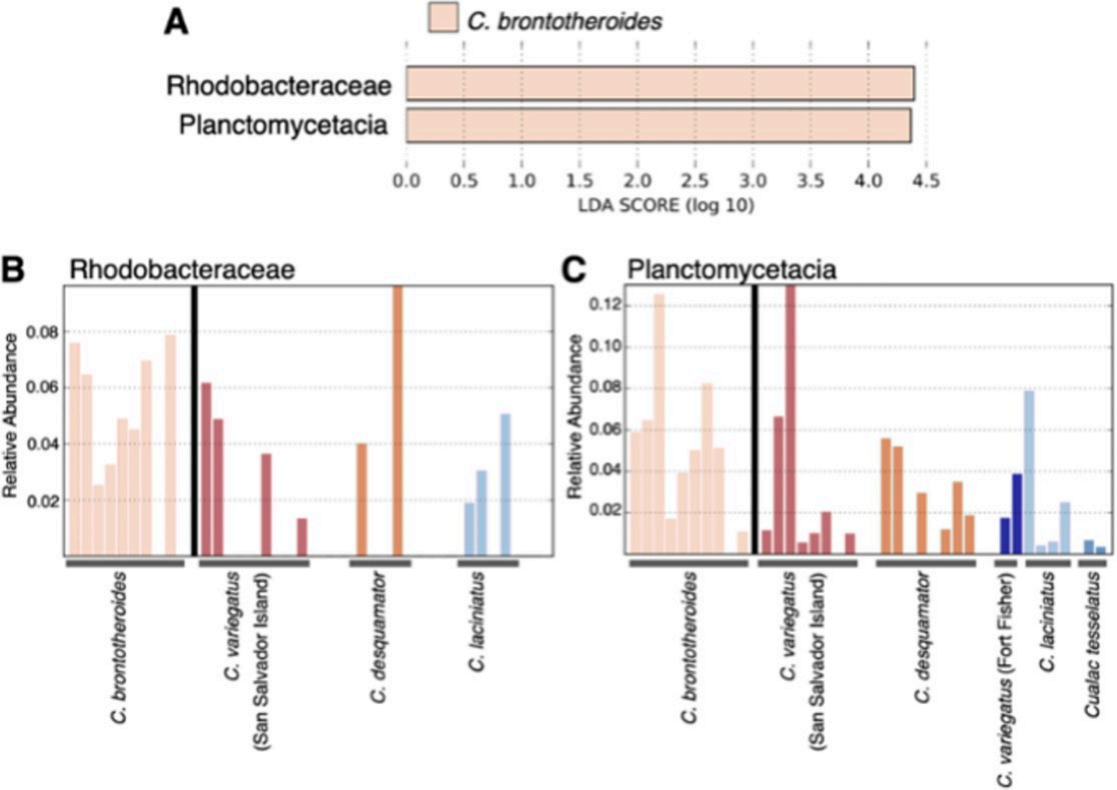
Linear discriminate analysis between *Cyprinodon brontotheroides* (molluscivore) and non-molluscivores. A) Log scores of the top two dominant loadings on the lefse discriminate axis separating molluscivores from all other pupfish samples. B) Relative abundance of the family *Rhodobacteraceae* and the class C) *Planctomycetacia* from all *Cyprinodon* gut microbiomes.

We found an excess of reads belonging to taxa within the family *Burkholderiaceae* in all lab-reared scale-eater individuals from two different lake populations relative to all other gut microbiome samples (Figs 3 and 4; linear discriminant analysis log score = 4.85). We also identified 108 taxa in the family *Burkholderiaceae* across all scale-eater gut microbiomes (S6 Fig). In addition, we found a deficiency of *Vibrionales*, *Vibrionaceae*, and *Vibrio* in these scale-eater individuals relative to all other gut samples (LDA log scores = -5.22, -5.22, and -5.08, respectively; Fig 4). Similarly, we found an excess of reads belonging to taxa in the family *Rhodobacteraceae* and class *Planctomycetacia* in the molluscivores relative to all other gut samples (Fig 5; LDA log scores of 4.39 and 4.37, respectively).

## Discussion

Using a common garden experiment, we show that differences in gut microbial diversity across *Cyprinodon* pupfish species largely reflect phylogenetic distance in support of phylosymbiosis [52], rather than the recent rapid evolution of novel trophic specialization as predicted by adaptive radiation theory. Our study is consistent with Ren et al. [53] who also found limited microbiome divergence and minimal associations with ecomorph in an adaptive radiation of Puerto Rican *Anolis* lizards, even within wild lizards. From our NMDS plot, we see clear differences between the generalists from San Salvador Island and the outgroup generalists, included the most closely extant genus *Cualac* spanning 11 Mya of evolutionary history (Fig 2). Similar studied found gut microbiome diversity to associate more strongly with geography than phylogeny [54] or a combination of geography, diet, and host phylogeny [55]. These emerging studies of microbiome divergence within adaptive radiations of hosts provide an important counterpoint to the classic expectation of rapid phenotypic diversification and speciation during adaptive radiation [18–21]. For example, a previous study found that craniofacial traits were diversifying up to 1,000 times faster on San Salvador Island than neighboring generalist pupfish populations, including many of the same populations analyzed for gut microbiota in this study [23].

One major caveat is that we did not examine the microbiota of wild-collected animals feeding on their diverse natural resources of macroalgae, scales, and snails. Scales form up to 50% of the diet in scale-eaters [27] and wild gut microbiome samples surely would have revealed more substantial differences in microbiome diversity and composition among generalist and specialist species on San Salvador Island. However, our goal with this common garden study using lab-reared animals spanning multiple generations and fed an identical generalist-type diet for one month was to uncover any genetically based microbiome differences in these taxa by eliminating environmental effects as much as possible. Pupfishes exhibit no parental care and deposit external eggs on the substrate so vertical transmission also appears highly unlikely (but see Satoh et al. [56] for a potential example of vertical transmission in a scale-eating cichlid). It is also possible that even second-generation lab-reared scale-eater colonies consumed more scales from their conspecific tankmates due to elevated behavioral aggression; however, aggression levels are comparable in both trophic specialists [57]. Furthermore, by including two lab-reared colonies of each generalist and specialist species on San Salvador from genetically differentiated and ecologically divergent environments of Crescent Pond and Osprey Lake [23, 28], we aimed to connect significant differences in microbiome composition observed in our specialist species to their specialized diets, rather than their lake environment or genetic background. These results from our ordination plots (Fig 2) are even more surprising because trophic specialists show very little genetic differentiation from San Salvador Island generalists (*F*_*st*_ = 0.1–0.3; 24, 29), yet we see differences in microbiome taxa and abundance across all three species from San Salvador Island and we identified community level differences based on diet from our PERMANOVA. In addition, there is a clear difference between the San Salvador Island pupfishes microbiomes and the generalist outgroup microbiomes, which can be best explained by phylogenetic distance and habitat differences of the host (Fig 2). This has been demonstrated by across multiple fish taxa where host habitat is the key determinant of gut microbiome composition [58]. Even though there were varying conditions throughout our experiment including captive generation (F0, F1, and F2), salinities ranging from 5–10 ppt, and temperatures ranging from 23–30°C, these environmental ranges were distributed haphazardly across our species and populations over the course of treatment period and likely had minimal impact on our conclusions; furthermore, all San Salvador Island populations were lab-reared at least through the F1 generation. Potentially, if we collected wild pupfish gut microbiomes, we may find further resolution from each location, especially between the two locations of San Salvador Island pupfishes (Crescent Pond and Osprey Lake). In addition, we did not detect a gut length difference among the generalist, molluscivore, and scale-eaters F2 pupfishes from San Salvador after controlling for size. We had expected to see shorter gut lengths in the two trophic specialists because of their shift in diet. In marine prickleback fishes (stichaeids), there are differences in gut length due to dietary diversity [59]. Overall, metabolic processes were the single most enriched category among all differentially expressed genes between these trophic specialists at the 8 dpf larval stage, accounting for 20% of differential expression [60], suggesting that gene expression differences may explain differences in microbial communities rather than gross anatomical differences.

### Enriched microbiota in scale-eating pupfish

Fish scales are composed of a deep layer that is mostly collagen type I [61]; therefore, we predicted that any adaptive microbes within the scale-eater gut would have collagen degrading properties. We found significant enrichment of one family, the *Burkholderiaceae* in both scale-eater populations (Figs 3, 4, and S5). *Burkholderiaceae* is a family of proteobacteria which contains many human and animal pathogens [62], plant and insect symbionts [63, 64], and can be found in soil, water, and polluted environments [65, 66]. Importantly, they also include some collagenase-producing bacteria, such as *Burkholderia pseudomallei* [UniProtKB–A3P3M6; 67], which is the causative agent of melioidosis in humans [68]. The significant shift in a major collagenase-producing group suggests the potential for an adaptive scale-eater microbiome, even in the absence of dietary scales (except perhaps for incidental aggression and ingestion of scales among tankmates).

In contrast to a microbiome study of the adaptive radiation of Tanganyikan cichlids [69], we found no evidence of *Clostridia* enrichment in scale-eaters nor a reduction of microbial diversity in this carnivorous species. This may be due to the very young 10 kya age of the scale-eating pupfish relative to the comparatively ancient 12 Mya Tanganyikan radiation and Perissodus scale-eating clade [27, 70].

### Enriched microbiota in molluscivore pupfish

We found enrichment of the families *Rhodobacteraceae* and *Planctomycetacia* within the molluscivore gut from both lake populations (Fig 5). Both of these taxa are present in the water controls, but not at the level present in the molluscivores (S7 Fig). However, these families have no clear role in anything related to mollusc digestion or even increased levels of protein, lipids, or chitin in the diet (due to some molluscivores specializing on ostracods during periods of abundance). Taxa from these taxonomic group are known to be found within aquatic environments [71, 72]. As noted by Simon et al. [71], marine microbes from the family *Rhodobacteraceae* play a crucial role in biogeochemical cycling, make up about 30% of bacterial communities within pelagic environments, and generally have a mutualistic relationship with eukaryotes providing vitamins to these groups. Both families are known for aquatic cellulose-decomposing taxa [73, 74], which suggests this microbiome shift may help more with macroalgae digestion rather than molluscs, despite previous observations that macroalgae forms the largest component of the generalist pupfish diet in the hypersaline lakes of San Salvador Island, Bahamas [27].

## Conclusion

Many studies have focused on understanding digestion and assimilation within a variety of vertebrates and invertebrates, but there is limited information about the cooperative process between the host intestine cells and gut microbiota, and their role in eco-evolutionary dynamics during rapid species diversification [10, 59, 75]. We found evidence for enrichment of taxa in the *Burkholderiaceae* family within the scale-eater microbiome (Fig 2), even when hosts were reared in identical environments on identical non-scale diets. However, it is still unknown to what extent this microbiome shift will improve digestion of the collagen found in scales, for example, as demonstrated for the gut fauna in the scale-eating khavalchor catfish [76]. Despite unique and highly specialized pupfish dietary adaptations within shared hypersaline lake habitats, overall gut microbial diversity did not follow the expected pattern of rapid diversification and divergence as observed in their hosts, calling into question how eco-evolutionary dynamics between host and symbiont proceed during adaptive radiation.

## Supporting information

S1 Fig Rarefaction for all 48 (16S) microbiome samples used in this study. Rarefaction curve constructed based on Amplicon Sequence Variant (ASVs), and samples with less than 6,000 reads (sequence depth) are shown with labels.
(TIF)

S2 Fig Scatter plot of the covariate (log standard length) and the outcome variable (log gut length) for all *Cyprinodon* pupfish species in our study. Closed circles represent the two specialists (scale-eater and molluscivore) and open circles represent generalists.
(TIF)

S3 Fig Total abundance of gut microbes across all *Cyprinodon* pupfish species used in this study. Thirty-two phyla of microbes represented across all gut microbiomes, not including controls.
(TIF)

S4 Fig A cluster dendrogram based on pupfish gut microbiome taxa using a Bray-Curtis distance (averaged). For the San Salvador Island samples only, individuals numbered as 1–5 represent Crescent Pond and 6–10 represent Osprey Lake. Scale = scale-eater, Moll = molluscivore, and Gen = generalist.
(TIF)

S5 Fig A cluster dendrogram based on pupfish gut microbiome taxa using a Bray-Curtis distance (averaged). For the San Salvador Island samples only, individuals numbered as 1–5 represent Crescent Pond and 6–10 represent Osprey Lake. Outgroup species to our study are in different shades of blue. Samples which did not cluster with the majority of the San Salvador Island samples as depicted in S4 Fig were removed from the analysis to determine if the same clustering pattern appeared.
(TIF)

S6 Fig Abundance counts of genera within the family *Burkholderiaceae* found in the Scale-eater pupfish gut microbiomes. Individuals numbered as 1–5 and 6–10 had parental colonies from Crescent Pond and Osprey Lake, respectively.
(TIF)

S7 Fig Abundance counts of *Rhodobacteraceae* and *Planctomycetacia* of *Cyprinodon* pupfishes gut microbiomes based on parental location and diet type along with controls. Lake 1 indicates Crescent Pond and Lake 2 represents Osprey Lake, both located on San Salvador Island in the Bahamas.
(TIF)

S1 Table Sample size, location, standard length, gut length, and relative gut length of *Cyprinodon* pupfish guts. (DOCX)

S2 Table Read counts. (DOCX)
