# Supplementary figures and images for: Minimal overall divergence of the gut microbiome in an adaptive radiation of Cyprinodon pupfishes despite potential adaptive enrichment for scale-eating
Source: PLoS One. 2022 Sep 16;17(9):e0273177. doi: 10.1371/journal.pone.0273177 (PMC9481044; doi:10.1371/journal.pone.0273177)

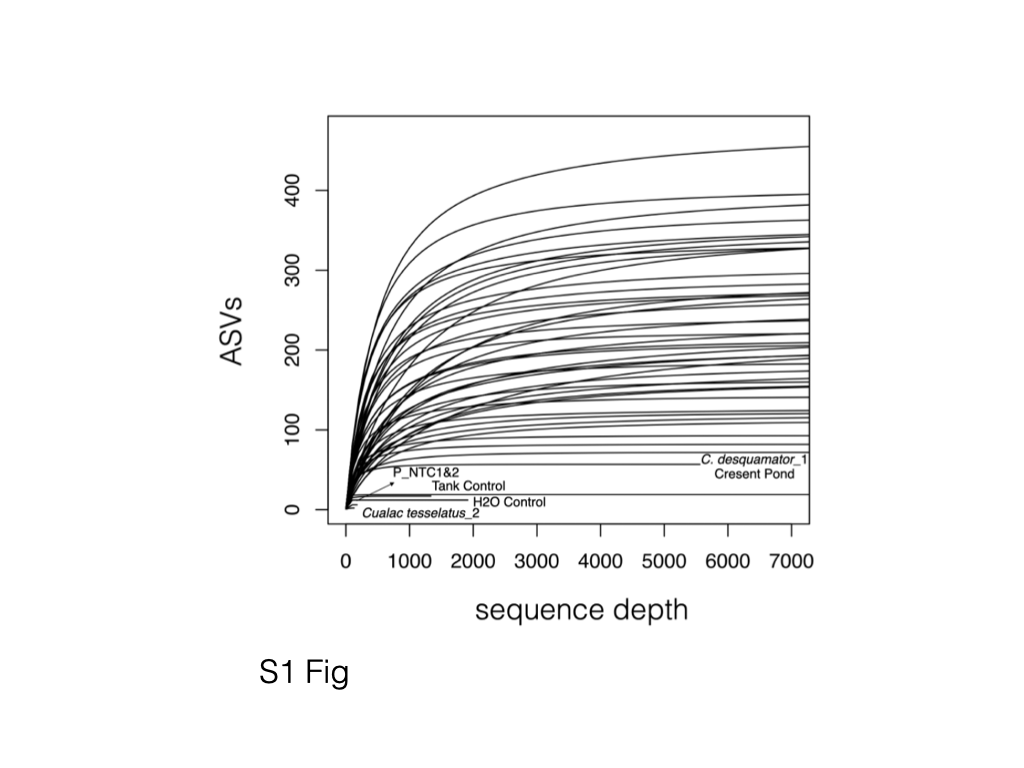

Supplement: S1 Fig — Rarefaction curve constructed based on Amplicon Sequence Variant (ASVs), and samples with less than 6,000 reads (sequence depth) are shown with labels. (TIF) [file pone.0273177.s001.tif]

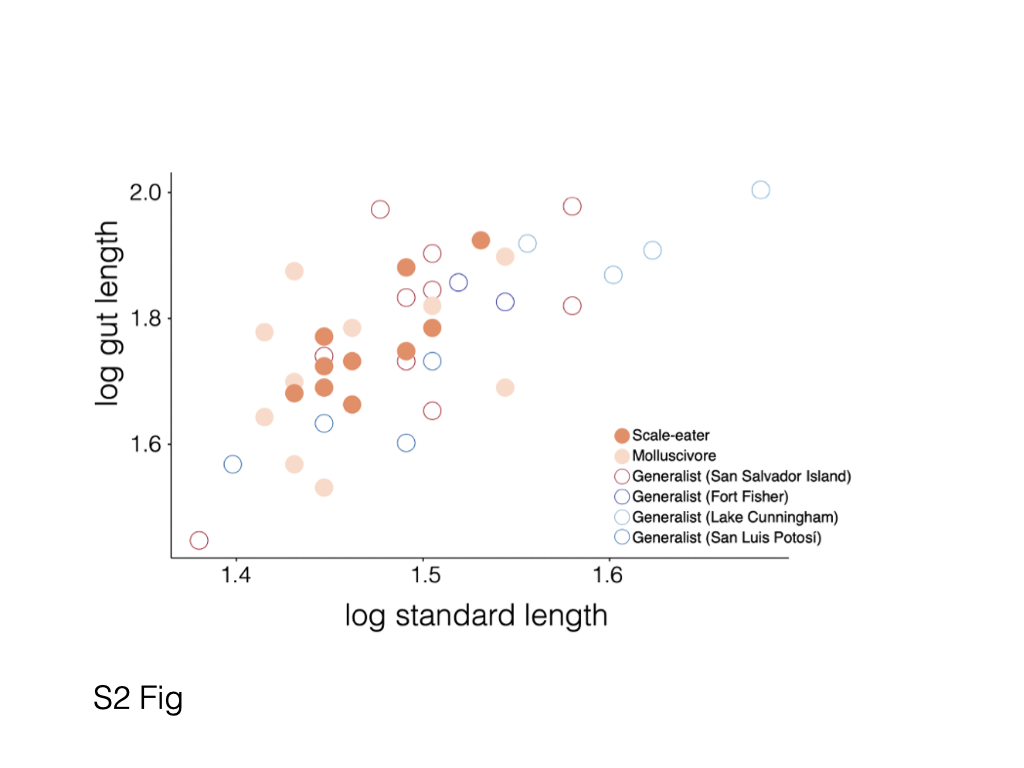

Supplement: S2 Fig — Closed circles represent the two specialists (scale-eater and molluscivore) and open circles represent generalists. (TIF) [file pone.0273177.s002.tif]

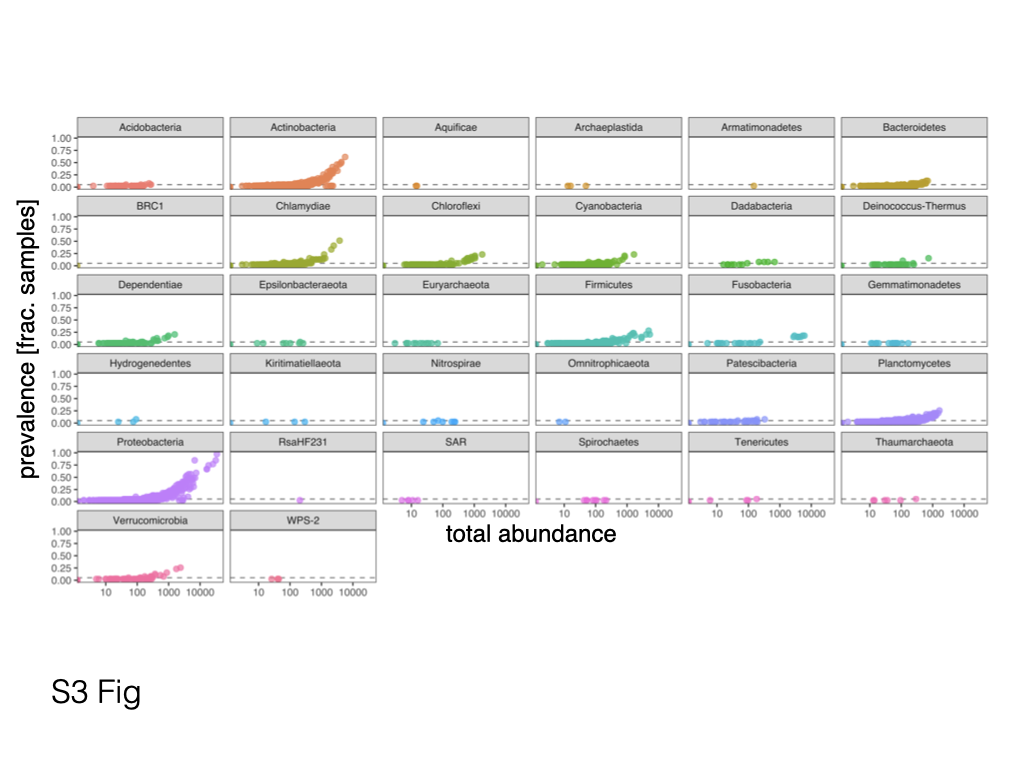

Supplement: S3 Fig — Thirty-two phyla of microbes represented across all gut microbiomes, not including controls. (TIF) [file pone.0273177.s003.tif]

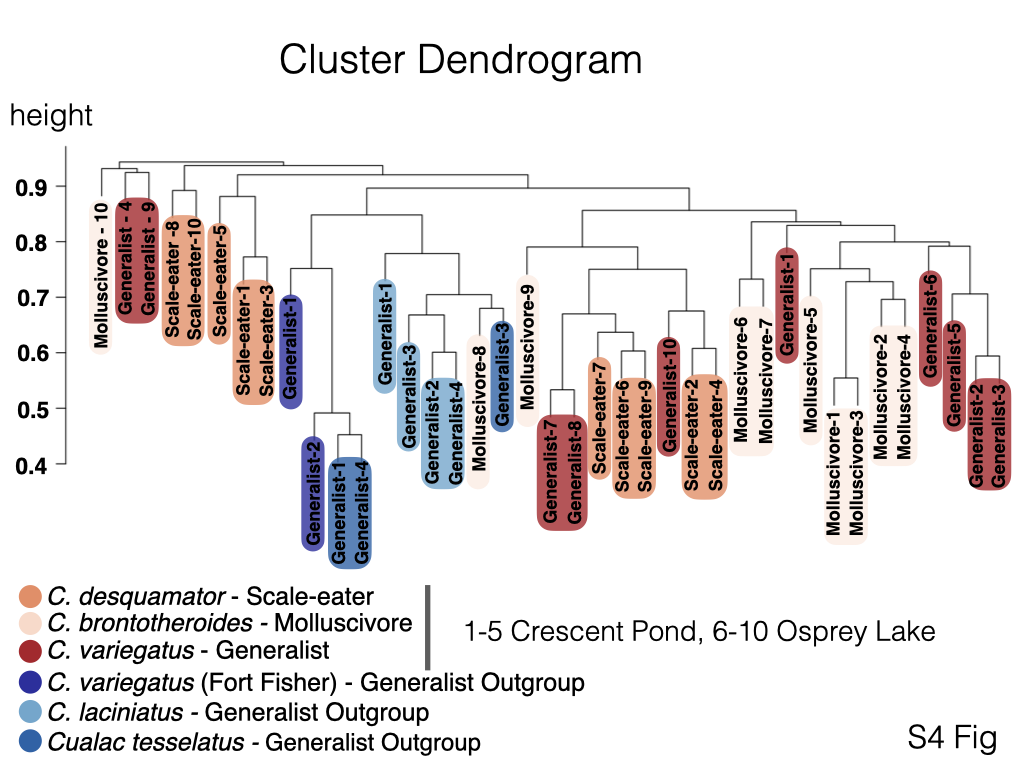

Supplement: S4 Fig — For the San Salvador Island samples only, individuals numbered as 1–5 represent Crescent Pond and 6–10 represent Osprey Lake. Scale = scale-eater, Moll = molluscivore, and Gen = generalist. (TIF) [file pone.0273177.s004.tif]

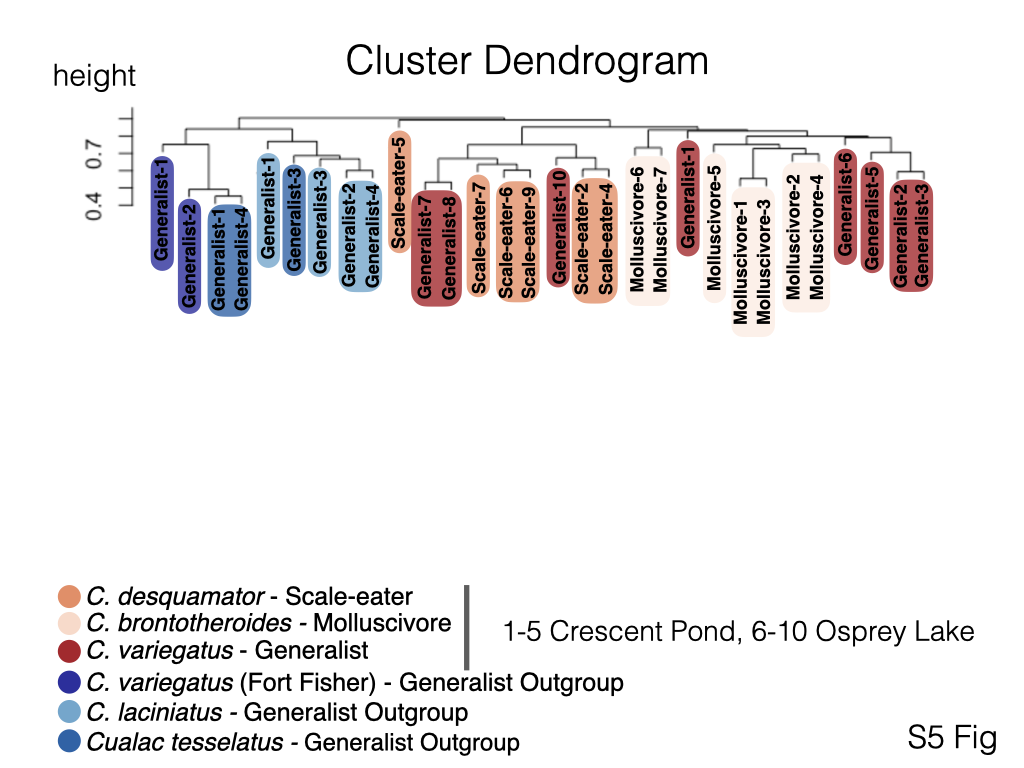

Supplement: S5 Fig — For the San Salvador Island samples only, individuals numbered as 1–5 represent Crescent Pond and 6–10 represent Osprey Lake. Outgroup species to our study are in different shades of blue. Samples which did not cluster with the majority of the San Salvador Island samples as depicted in S4 Fig were removed from the analysis to determine if the same clustering pattern appeared. (TIF) [file pone.0273177.s005.tif]

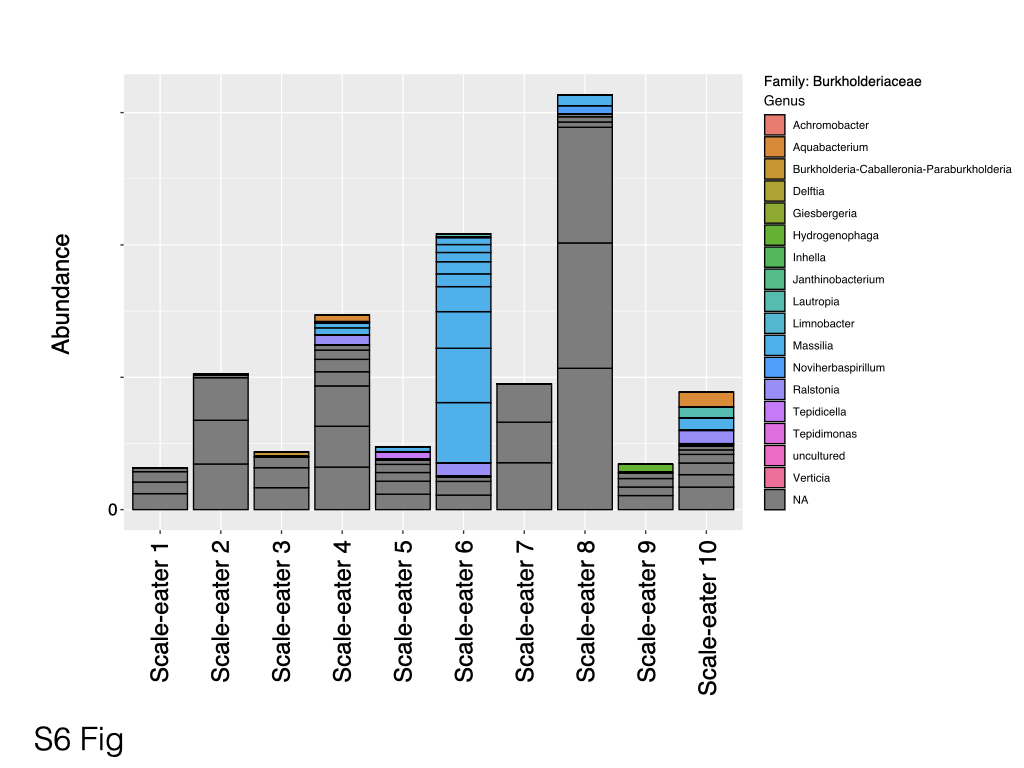

Supplement: S6 Fig — Individuals numbered as 1–5 and 6–10 had parental colonies from Crescent Pond and Osprey Lake, respectively. (TIF) [file pone.0273177.s006.tif]

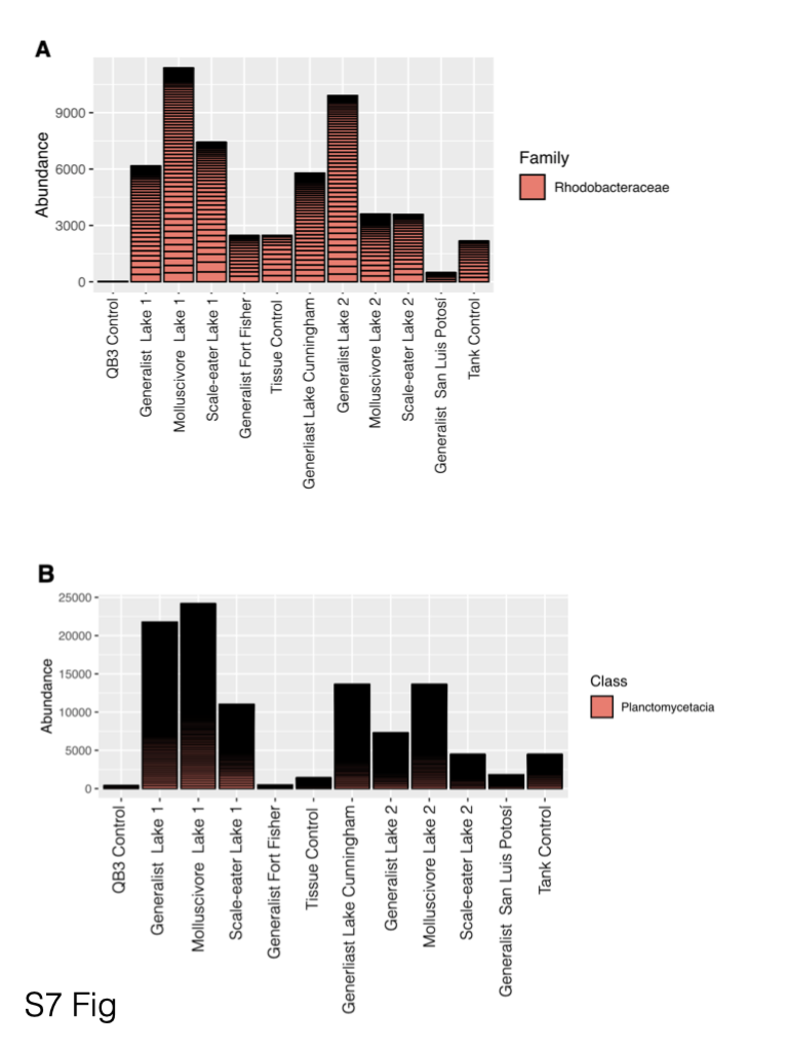

Supplement: S7 Fig — Lake 1 indicates Crescent Pond and Lake 2 represents Osprey Lake, both located on San Salvador Island in the Bahamas. (TIF) [file pone.0273177.s007.tif]
