# Supplementary material for: Minimal overall divergence of the gut microbiome in an adaptive radiation of Cyprinodon pupfishes despite potential adaptive enrichment for scale-eating
Source: PLoS One. 2022 Sep 16;17(9):e0273177. doi: 10.1371/journal.pone.0273177 (PMC9481044; doi:10.1371/journal.pone.0273177)
Supplement: S1 Table — (DOCX) [file pone.0273177.s008.docx]

| **S1 Table: Sample Size, Location, Standard Length, Gut Length, and Relative Gut Length of *Cyprinodon* pupfish guts** | | | | | | | | | |
| --- | --- | --- | --- | --- | --- | --- | --- | --- | --- |
| **Species name:** | **Sample size (n)** | **Location** | **Std. Length Range**  **(mm)** | **Std. Length Avg.** | **Std. Length S.D.** | **Gut Length Range**  **(mm)** | **Gut Length Avg.** | **Gut Length S.D.** | **Relative Gut Length Avg.** |
| ***Cyprinodon variegatus***  **Generalist** | 5 | Crescent Pond, San Salvador Island, Bahamas | 30-38 | 32.6 | 3.13 | 66-94 | 75.6 | 11.61 | 2.32 |
| ***Cyprinodon brontotheroides***  **Molluscivore** | 5 | Crescent Pond, San Salvador Island, Bahamas | 26-35 | 30.4 | 4.34 | 49-79 | 64.8 | 12.17 | 2.13 |
| ***Cyprinodon desquamator***  **Scale-eater** | 5 | Crescent Pond, San Salvador Island, Bahamas | 28-34 | 30.6 | 2.61 | 49-84 | 65.8 | 14.02 | 2.15 |
| ***Cyprinodon variegatus***  **Generalist** | 5 | Osprey Lake, San Salvador Island, Bahamas | 24-38 | 30.6 | 5.18 | 28-95 | 55.4 | 24.64 | 1.81 |
| ***Cyprinodon brontotheroides***  **Molluscivore** | 5 | Osprey Lake, San Salvador Island, Bahamas | 26-32 | 28 | 2.10 | 34-66 | 46.2 | 12.70 | 1.65 |
| ***Cyprinodon desquamator***  **Scale-eater** | 5 | Osprey Lake, San Salvador Island, Bahamas | 27-31 | 28.8 | 1.48 | 46-56 | 51.4 | 4.22 | 1.78 |
| ***Cyprinodon variegatus***  **Generalist** | 2 | Fort Fisher, North Carolina, USA | 33-35 | 34 | 1.14 | 67-72 | 69.5 | 3.54 | 2.04 |
| ***Cyprinodon laciniatus***  **Generalist** | 4 | Lake Cunningham, New Providence Island, Bahamas | 36-48 | 41.5 | 5.00 | 83-101 | 84.75 | 11.50 | 2.04 |
| ***Cualac tesselatus***  **Generalist** | 4 | San Luis Potosí, Mexico | 25-32 | 29 | 3.16 | 37-54 | 43.5 | 7.42 | 1.5 |
