# Supplementary material for: Minimal overall divergence of the gut microbiome in an adaptive radiation of Cyprinodon pupfishes despite potential adaptive enrichment for scale-eating
Source: PLoS One. 2022 Sep 16;17(9):e0273177. doi: 10.1371/journal.pone.0273177 (PMC9481044; doi:10.1371/journal.pone.0273177)
Supplement: S2 Table — (DOCX) [file pone.0273177.s009.docx]

| **S2 Table: Read Counts** | | |
| --- | --- | --- |
| **Sample ID** | **Number of Reads (input)** | **Number of Reads (non-chimeric)** |
| **P_NTC1** | 47 | 20 |
| **SLP_Ctesselatus_2** | 469 | 129 |
| **P_NTC2** | 251 | 176 |
| **OspreyLake_Cvariegatus_tank** | 3433 | 1334 |
| **H2O** | 2866 | 1916 |
| **CrescentPond_Cdesquamator_1_BOTH** | 16938 | 5560 |
| **P_POS** | 130311 | 8742 |
| **SLP_Ctesselatus_4** | 64007 | 12000 |
| **NC_Cvariegatus_2** | 90499 | 15200 |
| **SLP_Ctesselatus_1** | 100586 | 15299 |
| **OspreyLake_Cbrontotheroides_3_BOTH** | 125731 | 20896 |
| **LC_Claciniatus_4** | 187447 | 29622 |
| **SLP_Ctesselatus_3** | 106536 | 31008 |
| **CrescentPond_Cvariegatus_tank** | 114151 | 36440 |
| **LC_Claciniatus_2** | 167488 | 38727 |
| **NC_Cvariegatus_1_LIVER** | 163586 | 39508 |
| **LC_Claciniatus_3** | 195774 | 41299 |
| **OspreyLake_Cdesquamator_4_BOTH** | 330222 | 41382 |
| **OspreyLake_Cdesquamator_2_BOTH** | 201361 | 42532 |
| **NC_Cvariegatus_1** | 148071 | 43837 |
| **LC_Claciniatus_1** | 146701 | 44590 |
| **OspreyLake_Cvariegatus_5_BOTH** | 200654 | 46348 |
| **CrescentPond_Cdesquamator_3_BOTH** | 261256 | 48502 |
| **CrescentPond_Cbrontotheroides_1_BOTH** | 203954 | 52047 |
| **CrescentPond_Cdesquamator_2_BOTH** | 243149 | 52244 |
| **CrescentPond_Cvariegatus_5_BOTH** | 230860 | 52309 |
| **CrescentPond_Cbrontotheroides_3_BOTH** | 222127 | 54438 |
| **OspreyLake_Cbrontotheroides_4_BOTH** | 302388 | 54841 |
| **CrescentPond_Cvariegatus_3_BOTH** | 196082 | 55839 |
| **CrescentPond_Cbrontotheroides_5_BOTH** | 210567 | 56836 |
| **NC_Cvariegatus_2_LIVER** | 111440 | 59562 |
| **CrescentPond_Cvariegatus_2_BOTH** | 316634 | 73318 |
| **CrescentPond_Cvariegatus_1_BOTH** | 428036 | 78415 |
| **CrescentPond_Cbrontotheroides_4_BOTH** | 240558 | 79695 |
| **OspreyLake_Cdesquamator_1_BOTH** | 254716 | 83178 |
| **OspreyLake_Cdesquamator_3_BOTH** | 236572 | 88224 |
| **OspreyLake_Cbrontotheroides_1_BOTH** | 324187 | 91701 |
| **OspreyLake_Cbrontotheroides_2_BOTH** | 359079 | 97676 |
| **OspreyLake_Cdesquamator_5_BOTH** | 332246 | 97944 |
| **OspreyLake_Cvariegatus_2_BOTH** | 394576 | 105642 |
| **OspreyLake_Cbrontotheroides_5_BOTH** | 358106 | 109573 |
| **CrescentPond_Cvariegatus_4_BOTH** | 493304 | 120672 |
| **OspreyLake_Cvariegatus_4_BOTH** | 277417 | 126381 |
| **CrescentPond_Cbrontotheroides_2_BOTH** | 455758 | 132768 |
| **OspreyLake_Cvariegatus_3_BOTH** | 444809 | 139478 |
| **CrescentPond_Cdesquamator_4_BOTH** | 421807 | 161716 |
| **OspreyLake_Cvariegatus_1_BOTH** | 524547 | 196367 |
| **CrescentPond_Cdesquamator_5_BOTH** | 810843 | 271427 |
